# Hand Posture Modulates Perceived Tactile Distance

**DOI:** 10.1038/s41598-017-08797-y

**Published:** 2017-08-29

**Authors:** Matthew R. Longo

**Affiliations:** Department of Psychological Sciences, Birkbeck, University of London, London, England

## Abstract

A growing literature shows that body posture modulates the perception of touch, as well as somatosensory processing more widely. In this study, I investigated the effects of changes in the internal postural configuration of the hand on the perceived distance between touches. In two experiments participants positioned their hand in two postures, with the fingers splayed (*Apart* posture) or pressed together (*Together* posture). In Experiment 1, participants made forced-choice judgments of which of two tactile distances felt bigger, one oriented with the proximal-distal hand axis (*Along* orientation) and one oriented with the medio-lateral hand axis (*Across* orientation). In Experiment 2, participants made verbal estimates of the absolute distance between a single pair of touches, in one of the two orientations. Consistent with previous results, there was a clear bias to perceive distances in the across orientation as larger than those in the along orientation. Perceived tactile distance was also modulated by posture, with increased judgments in both orientations when the fingers were splayed. These results show that changes in the internal posture of the hand modulate the perceived distance between touches on the hand, and add to a growing literature showing postural modulation of touch.

## Introduction

Several forms of somatosensory perception require that immediate sensory signals be combined with higher-level representations of the body^1^. Recent research investigating these body representations has revealed that they feature large spatial distortions, both in the case of position sense^2–8^ and tactile distance perception^9–19^. In both of these domains there are substantial biases for distance oriented with the medio-lateral axis of the limbs to be overestimated in comparison to distances oriented in the proximo-distal axis^20^. Other studies have found that changes to the internal posture of the hand (i.e., the relative position of the parts of the hand with respect to each other) alter the organization of body maps in somatosensory cortex^21–23^. I recently found that changing the internal posture of the hand leads to rapid changes in the size of perceptual maps of the hand underlying position sense^24^. The present study thus investigated whether changes in hand posture produce similar changes in perceived tactile distance.

## Perceptual Distortions of Tactile Distance

In his classic investigations of touch, Weber^25^ observed that as he moved the two points of a compass across his skin it felt as if the points became farther apart as they moved from a region of relatively low sensitivity (e.g., the forearm) to a region of relatively high sensitivity (e.g., the hand). This effect, commonly known as *Weber’s illusion*, has been replicated in many subsequent studies^10, 12, 18, 26, 27^, which have found a generally systematic relation between perceived tactile distance and tactile spatial sensitivity, as if the familiar distortions of the somatosensory homunculus^28^ are preserved in perception.

Similar perceptual distortions have also been found comparing stimuli in different orientations on a single skin surface. In general, stimuli oriented across the medio-lateral axis of the arms are perceived as larger than stimuli oriented along the proximo-distal limb axis^9, 13, 15–18^. Similar biases have also been found on the legs^9^ and the face^29^. Longo and Haggard^13^ suggested that both the classic Weber’s illusion and the orientational anisotropies in perceived tactile distance could result from the geometry of receptive fields (RFs) of neurons in somatosensory cortex. RFs are smaller on highly sensitive skin surfaces than on less sensitive surfaces^30, 31^ and are generally oval-shaped on the limbs, elongated along the proximo-distal limb axis^32, 33^.

The results described in the previous two paragraphs show that perceived tactile distance is shaped by the low-level organization of the somatosensory system. Other results, however, show that it is also modulated by higher-level representations of the body. For example, visual magnification of the forearm leads to a reduction of the baseline magnitude of Weber’s illusion comparing stimuli on the forearm and hand^10^. Other studies have shown analogous modulations of perceived tactile distance by modulations of the body induced by proprioceptive illusions^11^, auditory experience^14, 34^, vision of the body^16^, categorical segmentation of the body at joints^17, 35^, and tool use^15, 36, 37^. Thus, the perception of tactile distance is shaped both from the bottom-up by the basic organization of the somatosensory system, and from the top-down by multisensory representations of body size and shape.

## Postural effects on touch

Several lines of research have shown that changes in body posture modulate the processing of touch. For example, in the classic ‘crossed hands deficit’, the ability to discriminate the temporal order of two touches, one on each hand, is dramatically impaired when the limbs are crossed^38–42^. The perceived location of touch appears to be coded based on the usual location of the limb, rather than it’s actual location, for the first 80–100 ms following touch^43^. Similarly, crossing the arms over the body midline reduces the perceived intensity of body tactile and painful stimuli^44^. In contrast, crossing individual fingers seems not to lead to updating of posture, even with delays as long as 700 ms^45^, as seen in the classic ‘Aristotle illusion’ in which an object placed between crossed fingertips is perceived to be two distinct objects^46^. In another study, interleaving the fingers of the two hands impaired judgments of which hand was touched, but not of the identity of the touched finger^47^. This pattern suggests that hand identity, but not finger identity, is coded based on external spatial locations, though for a different view see ref. 48. Similarly, Tamè and colleagues^49^ found that patterns of interference between homologous fingers were modulated by the congruency in posture between the two hands.

Other studies have found that limb posture modulates tactile impairments following stroke. For example, Medina and Rapp^50^ described a patient who experienced bilateral sensations on both the right and left hands when touch was applied only to the left hand, a condition known as ‘synchiria’. The strength of synchiria was systematically modulated by the posture of the limbs in space, becoming stronger as the limbs were moved towards the contralesional right hemispace. Similarly, several studies of tactile extinction, in which patients fail to perceive touch on the contralesional hand when presented simultaneously with touch on the ipsilesional hand, have found that the strength of extinction is modulated by the posture of the limbs^51–57^.

Neuroimaging studies have revealed that changes in the internal postural configuration of the hand modulates processing in somatosensory cortex. Hamada and Suzuki^21, 22^ used magnetoencepholography (MEG) to investigate activations to electrical stimuli applied to the thumb and index finger when the hand was ‘open’ (with fingers spread apart) or ‘closed’ (with the fingers close, but not touching). This postural change modulated both the pattern of interactions between the two fingers^21^ and the distance between the dipoles for the two digits in secondary somatosensory cortex^22^. These results suggest that changes in the internal posture of the hand produce rapid modulations of low-level somatotopic maps. Similarly, Stavrinou and colleagues^23^ taped together the four fingers of participants’ hands, inducing an experimental form of ‘syndactyly’, analogous to surgical interventions performed in monkeys^58^. Half an hour following taping, the distance between MEG dipoles for the index and little fingers was reduced relative to baseline, suggesting that the representations of the fingers had become less distinct.

Two recent behavioural studies have found that spreading the fingers apart reduces mislocalisations between the fingers^59, 60^, consistent with the above results suggesting that an open hand posture makes digit representations more distinct. Similarly, Tamè and colleagues^60^ also found that spreading the fingers led to an increase in the number of unstimulated fingers in-between two stimulated fingers, a classic measure of structural body representations^61^. Most directly relevant to the current study, I recently found that implicit perceptual maps underlying position sense are modulated by hand posture^24^. Specifically, when the fingers were splayed, the maps were expanded in size compared to when the fingers were pressed together, resulting in an increase in the overestimation of hand width and a decrease in the underestimation of finger length. In contrast, no modulation of map size was apparent in a previous study comparing two conditions which differed in terms of the rotation of the hand relative to the torso^2^. Thus, it is not changes in posture in general that affected hand representation, but specifically changes in the internal posture of the hand, that is in the posture of the parts of the hand relative to each other, rather than to the larger spatial structure of the body.

## The present study

This study investigated the effects of internal hand posture on the perception of tactile distance. Given the results described above showing that an open hand posture makes the representations of the fingers more distinct, I predicted that it would similarly lead to an increase in perceived tactile distance across the width of the hand. Participants placed their left hands into two postures, with the fingers either pressed together or splayed apart. In Experiment 1, participants made two-alternative forced-choice (2AFC) about which of two tactile distances felt larger, one oriented with the medio-lateral hand axis and the other with the proximo-distal axis. Perceptual bias in the two postures was assessed by identifying the ratio between the two stimuli at which they were subjectively perceived as equal. In Experiment 2, participants made verbal size estimates of the extent of single tactile distances.

## Experiment 1 – Forced-Choice Judgments

### Method

#### Participants

Eighteen members of the Birkbeck community (nine women) between 17 and 41 years of age (*M*: 30.7 years) participated. All participants but one were right-handed as assessed by the Edinburgh Inventory^62^ (*M*: 75.94). All participants gave written informed consent before participating. Procedures were approved by the Department of Psychological Sciences ethics committee at Birkbeck, University of London, and were in accordance with the principles of the Declaration of Helsinki.

#### Procedures

The stimuli were wooden sticks which tapered to a point (~1mm) but were not sharp, similar to those we have used in previous studies^13, 16, 19, 29, 63, 64^. Pairs of sticks were mounted in foamboard, separated by 20, 30, or 40 mm. On each trial the participant was touched on the dorsum of the left hand with two tactile distances in sequence, one oriented with the mediolateral hand axis and the other oriented with the proximodistal hand axis. Each touch was applied manually by the experimenter for approximately one second with an inter-stimulus interval of approximately one second. Manual delivery of stimuli has the drawback that the duration, inter-stimulus interval, and pressure of stimuli are not exactly matched from trial to trial. Nevertheless, such stimulation was preferred given that it produces a clear and firm tactile sensation, which is difficult to create with other stimuli such as solenoid tappers. Moreover, manual delivery makes it easy to jitter the exact location of stimulation from trial-to-trial in order to avoid adaptation or sensitization of specific areas of skin.

Participants made unspeeded verbal 2AFC judgments of whether the first or the second distance felt bigger. Across trials, there were five different pairs of distances, varying in the ratio of the distances in the across and along orientations (across/along): 20/40 mm, 20/30 mm, 30/30 mm, 30/20 mm, 40/20 mm.

**Figure 1:**
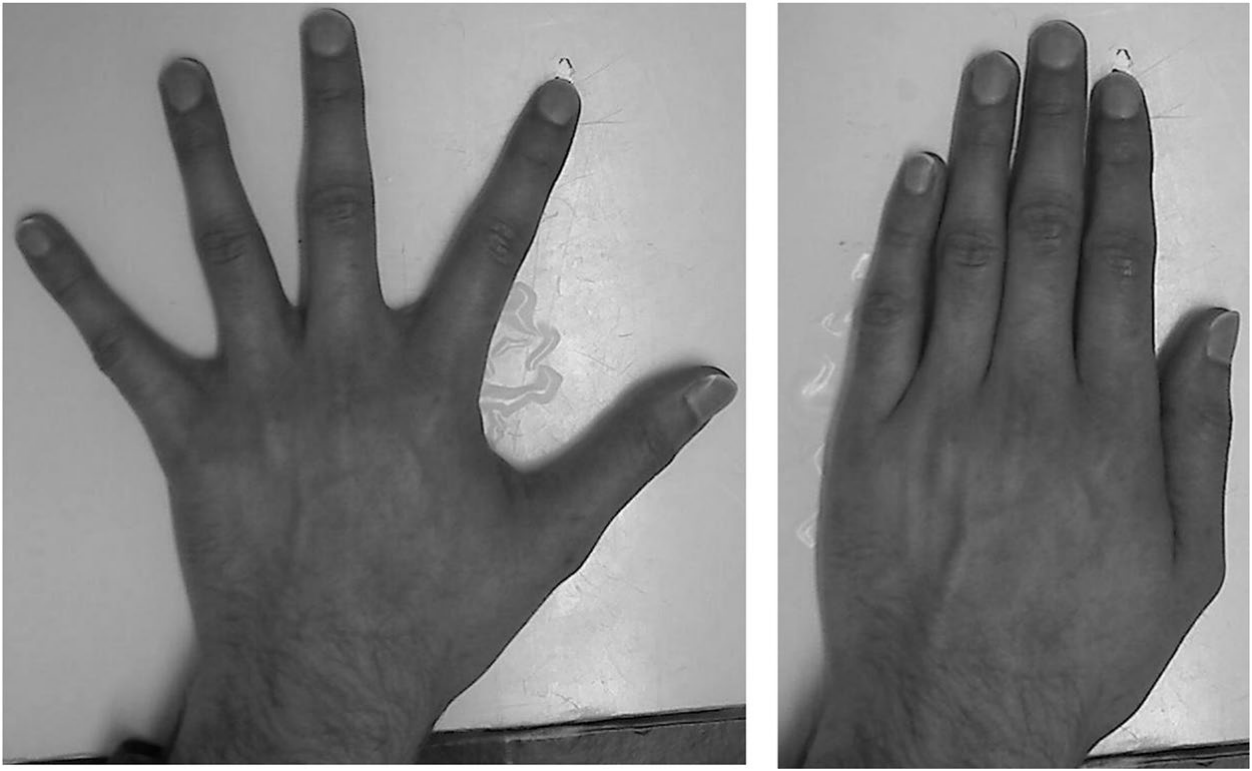
The two postures used. In the *Apart* posture (left panel) the participant was asked to hold their hand with the fingers spread as far apart as would be comfortable to hold throughout the block. In the *Together* posture (right panel), they were asked to hold their hand with the fingers pressed together.

Across blocks, the internal posture of the participant’s hand was manipulated, as in my recent study measuring proprioceptive hand maps^24^. In each case, the participant sat at a table with their left hand resting comfortably on the table, with the palm facing down. In the *Together* posture, the participant was asked to place the fingers of their hand together (Fig. 1, left panel). In the *Apart* posture, the participant was asked to spread the fingers apart by the maximum amount that would be comfortable to hold throughout the entire block (Fig. 1, right panel).

There were four blocks of trials, two of each hand posture. The order of the blocks was counterbalanced in an ABBA fashion, with the first block being counterbalanced across participants. Each block consisted of 40 trials, consisting of eight repetitions of each of the five trials types. Within these eight repetitions the order of the across and along stimuli were counterbalanced. The 40 trials within each block were presented in random order. Participants were allowed to take a short break between blocks, and were blindfolded throughout the experiment.

#### Analysis

For each trial type, the proportion of trials in which the ‘across’ distance was judged as larger than the ‘along’ distance was calculated. These proportions were analyzed as a function of the ratio of the size of the across and along distances, plotted using a logarithmic scale to produce a symmetric distribution around a ratio of 1 (i.e., the ratio at which the two distances are actually the same size). Cumulative Gaussian functions were fit to the data from each participant using maximum-likelihood estimation with the Palmedes toolbox^65^ for MATLAB (Mathworks, Natick, MA).

The criteria for exclusion of participants was if the psychometric function had an *R* ^2^ lower than 0.5 in either condition, as in other recent studies from our lab using this paradigm^29, 63^. In fact, however, good fit was obtained in all cases, so no participants were excluded.

The psychometric functions fit to the data are characterized by two parameters, the mean and the slope (i.e., 1/SD). The mean of the Gaussian indicates where it crosses 0.5 on the y-axis, and corresponds to the point-of-subjective-equality (PSE), the ratio between the across and along distances at which they are perceived as being equally far apart. If there were no perceptual bias, PSEs should on average equal 1; that is, the distances should be perceived as the same size when they actually are the same size. If there were a bias to perceive along distances as farther apart than across one, then PSEs should on average be larger than 1 (i.e., the across distance should need to be larger than the along one for them to be perceived as equal). In contrast, if there were a bias to perceive across distances as farther apart than along ones, then PSEs should on average be less than 1 (i.e., the along distance should need to be larger than the across one for them to be perceived as equal). Studies using this paradigm have consistently found PSEs to be less than 1, indicating a bias to perceive across distances on the hand dorsum as farther apart than along ones^13, 16, 17, 29, 36, 63^. The second parameter, the slope (the inverse of the standard deviation) reflects the steepness of the psychometric function. Large values of the slope indicate precise judgments.

To assess anisotropy in each posture, one-sample t-tests were used to compare mean PSEs to a ratio of 1. To compare anisotropy in the two postures, a paired t-test was used. Because the PSE is defined as a ratio of two distances, they were log-transformed before t-tests were performed. Slopes in the two postures were also compared using a paired t-test. In addition, performance in the two postures was compared using a 5 × 2 repeated-measures analysis of variance (ANOVA), including ratio (0.5, 0.67, 1, 1.5, 2) and posture (Together, Apart) as factors. Where Mauchley’s test indicated a violation of the sphericity assumption, the Greenhouse-Geisser correction was applied.

As measures of effect size, Cohen’s *d* is provided for one-sample t-tests, *d*_*z*_ for paired t-tests, and η_p_^2^ for *F*-tests.

## Results and Discussion

**Figure 2:**
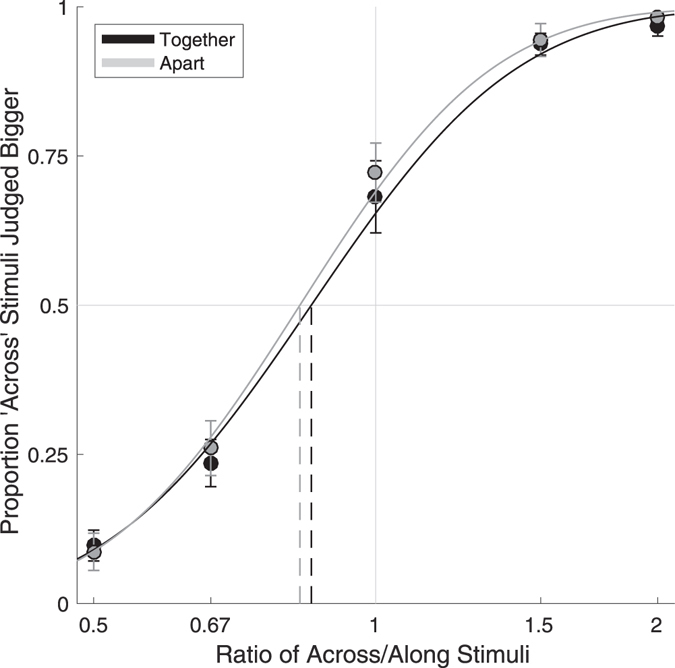
Results of Experiment 1. Data from the Curves fit to data are cumulative Gaussian functions. The dashed vertical lines indicate PSEs (i.e., where each curve crosses 0.5). Clear anisotropy was apparent in both conditions (i.e., PSEs are less than 1), with distances oriented across the hand perceived as larger than those oriented along the hand. However, there was no difference in the magnitude of anisotropy in the two postures. Error bars are one standard error.

The results of Experiment 1 are shown in Fig. 2. *R* ^2^ values indicated good fit to the data, with psychometric functions accounting for an average of 95.7% (SD: 5.8%) of the between-condition variance in the Together posture and 96.8% (SD: 3.0%) in the Apart posture. Clear anisotropies were apparent both in the together posture (Mean PSE: 0.844), *t*(17) = −3.60, *p* < 0.005, Cohen’s *d* = 0.849, and in the apart posture (Mean PSE: 0.820), *t*(17) = −4.17, *p* < 0.001, Cohen’s *d* = 0.982. Critically, however, the magnitude of anisotropy did not differ between the two postures, *t*(17) = 1.17, *n*.*s*., *d*_*z*_ = 0.276. There was a strong correlation between PSEs in the two postures, *r*(16) = 0.803, *p* < 0.0001. There was also no significant difference in the slopes of psychometric functions between the two postures, *t*(17) = 0.68, *n*.*s*., *d*_*z*_ = 0.160.

An ANOVA on the percentage of ‘across’ responses across conditions revealed a significant main effect of the ratio between the across and along stimuli, *F*(2.22, 37.68) = 243.57, *p* < 0.0001, η_p_^2^ = 0.935, but no main effect of posture, *F*(1, 17) = 1.02, *n*.*s*., η_p_^2^ = 0.057, and no interaction between ratio and posture, *F*(4, 68) = 0.35, *n*.*s*., η_p_^2^ = 0.020.

These results replicate the anisotropy for tactile distance perception on the hand dorsum which has been reported previously^9, 13, 15–17, 29, 36^, with distances oriented across the width of the hand being perceived as larger than distances oriented along the length of the hand. The magnitude of this anisotropy, however, did not appear to be modulated by hand posture. These results thus provide no evidence that hand posture modulates the perception of tactile distance. A limitation of this experiment, however, is that because it assessed the relative perception of stimuli in the two orientations, it would not be able to identify isotropic changes in perceived tactile distance. That is, if posture produced similar changes in both to tactile distances in both the across and along posture, no apparent change would have been found in this experiment. In the case of proprioceptive perceptual maps, spreading the fingers apart produced increases in perceived distances in both orientations^24^. Thus, I ran a second experiment in which participants made absolute estimates of the size of individual tactile distances in either the across or along orientations.

## Experiment 2 – Absolute Size Judgments

### Method

#### Participants

Sixteen members of the Birkbeck community (nine women) between 22 and 45 years of age (*M*: 30.6 years) participated. All gave written informed consent before participating. Testing started on one additional participant, but was stopped midway through because he reported feeling only a single touch on a large majority of trials.

#### Procedures

Stimuli were identical to those in Experiment 1. On each trial, the participant was touched on the dorsum of their left hand by a single tactile distance, which lasted approximately one second. Participants made unspeeded verbal judgments of the perceived distance between the two touches by giving a number in cm. Participants were allowed to respond using inches if they were more comfortable doing so (two participants responded in inches). Participants were instructed to be as precise as possible in their judgments and to consider using decimal responses (e.g., 2.4 cm rather than just 2 cm). They were allowed to give a response of 0 cm if they felt only one touch.

As in Experiment 1, there were four blocks, two of each posture, counterbalanced in ABBA fashion with the first posture counterbalanced across participants. Each blocks consisted of 48 trials, including eight repetitions of each combination of orientation (across, along) and stimulus size (20, 30, 40 mm), in random order. There were thus 192 trials in total. Participants were allowed to take a short break between blocks, and were blindfolded throughout the experiment.

#### Analysis

For each participant, we identified outlier trials in which the participant’s response was more than 3 standard deviations from their average response for distances of that size. Overall, 0.39% of trials were excluded as outliers.

## Results and Discussion

**Figure 3:**
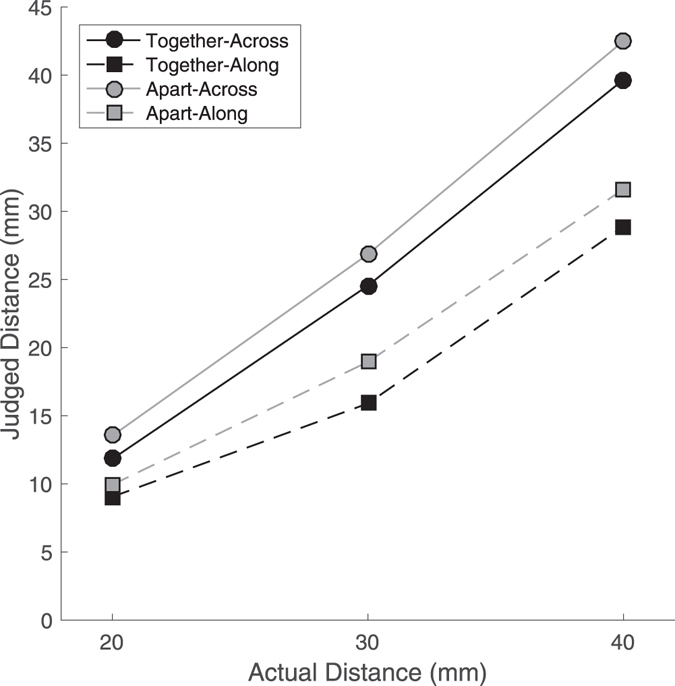
Results from Experiment 2, showing judged distance as a function of actual distance for each condition. As in Experiment 1, across distances were judged as larger than along distances. Critically, there was also an effect of posture. Distances in both orientations were judged as larger when the hand was in the Apart posture than when it was in the Together posture.

The results are shown in Fig. 3. Perceived distance increased monotonically with actual distance in all conditions. Linear regressions fit to individual participant data collapsed across postures showed excellent linear fit accounting for 98.3% (SD: 0.02%) of the between stimulus variance in the across orientation and 95.8% (SD: 0.06%) in the along orientation. There was a significant main effect of stimulus size, *F*(1.04, 15.53) = 22.47, *p* < 0.0005, η_p_^2^ = 0.600. In addition, there was a main effect of orientation, *F*(1, 15) = 31.82, *p* < 0.0001, η_p_^2^ = 0.0680, with distances in the across orientation judged as larger than those in the along orientation. Most importantly, there was a significant main effect of posture, *F*(1, 15) = 10.72, *p* < 0.01, η_p_^2^ = 0.417, with distances judged as larger with the hand in the apart posture than in the together posture. Follow-up t-tests using Holm-Bonferroni correction for multiple comparisons indicated that judged distances were larger in the apart than in the together posture for both across stimuli, *t*(15) = 2.35, *p* < 0.05, *d*_*z*_ = 0.588, and for along stimuli, *t*(15) = 3.28, *p* < 0.01, *d*_*z*_ = 0.820.

There was a significant interaction of stimulus size and orientation, *F*(2, 30) = 13.00, *p* < 0.0001, η_p_^2^ = 0.464, with the difference between the two orientations increasing with stimulus size. There were, however, no significant interactions involving posture (all *p*’s > 0.45).

## General Discussion

The present results show that changes in the internal posture of the hand do not alter the perception of the *relative* distance between pairs of touches in the across vs. along orientation (Experiment 1), but do lead to absolute increases in perceived tactile distance in both orientations (Experiment 2). This modulation by changes in the internal postural configuration of the hand is in contrast to previous results showing that *rotation* of the entire hand does appear to modulate perceived tactile distance^13^. These results contribute to a growing literature showing that body posture modulates the perception of touch^38–50, 60, 66–72^.

Independent of hand posture, there was a clear bias to overestimate distances oriented across the width of the hand compared to those oriented along the length of the hand. This was apparent both for forced-choice judgments (Experiment 1) and absolute size estimates of individual stimuli (Experiment 2). These results add to a growing literature showing large anisotropies of perceived tactile distance on the arms^9, 13, 15–17, 29, 36^, as well as on the leg^9^ and face^29^. This pattern mirrors lower-level aspects of the organization of the somatosensory system, such as the greater tactile acuity in the medio-lateral limb axis^25, 73^ and the fact that RFs of somatosensory neurons are generally oval-shaped with the long axis aligned with the proximo-distal limb axis^32, 33^.

The results of the present study investigating tactile distance perception are similar to those of a recent study showing the implicit hand maps underlying position sense^24^. In that study, I found that splaying the fingers led to an increase in the size of perceptual hand maps in both the proximo-distal axis (indexed by the distance between the knuckle and tip of each finger) and the medio-lateral axis (indexed by the distance between pairs of knuckles). The present results showing clear increases in perceived tactile distance in both orientations with fingers splayed is clearly consistent with that result. Broadly similar distortions are found for both position sense^2, 4, 74, 75^ and tactile distance perception^9, 13, 15, 16^, with clear overestimation of hand width relative to length in both cases. Perceptual distortions in both position sense and tactile distance perception parallel these characteristics of the somatosensory system, but are smaller in magnitude than would be expected by, for example, RF size alone^10, 20^. Thus, similar distortions are found in both position sense and tactile distance perception and they are both similarly modulated by internal hand posture.

What changes in somatosensory processing lead to the present results? Several studies using MEG have found that splaying the fingers leads to an increase in the distance between dipoles for touch on different fingers^21–23^. These results suggest that an open posture, such as the *apart* condition in the present study, leads to an increase in the distinctiveness of different parts of the hand. Thus, the whole hand may essentially be represented as larger when the fingers are splayed, potentially leading to the increase in perceived tactile distance described here. When the fingers are pressed together, the hand may be represented more as a single functional unit, while with fingers splayed it may be conceived as a collection of distinct parts. This interpretation is consistent with the recent finding of Tamè and colleagues^60^ that splaying the fingers leads to an increase in the perceived number of fingers judged as ‘in-between’ two stimulated fingers. Such changes with hand posture may relate to different functional modes of hand use, such as the classic distinction between power grips in which the fingers work as a single units vs. precision grips in which the fingers work more independently^76^.
